# Stage-Associated Reorganization of Epidural ECoG Functional Networks During Pigeon Homing Flight

**DOI:** 10.3390/ani16132025

**Published:** 2026-07-02

**Authors:** Fuli Jin, Yue Qin, Youtang Gao, Yanna Ping, Xiaomi Qi, Dongyun Wang, Xinyu Liu

**Affiliations:** 1 School of Electronic Information, Huanghuai University, Zhumadian 463000, China; jinfuli@huanghuai.edu.cn (F.J.); gaoyoutang@163.com (Y.G.); qixiaomin110001@126.com (X.Q.); 2 Henan Engineering Research Center of Intelligent Human-Machine Interaction Equipment, Huanghuai University, Zhumadian 463000, China; pingyanna@huanghuai.edu.cn (Y.P.); wangdongyun@huanghuai.edu.cn (D.W.); 3 School of Intelligent Manufacturing, Huanghuai University, Zhumadian 463000, China; lunaqin0630@163.com; 4 School of Electronic and Information, Zhongyuan University of Technology, Zhengzhou 450007, China

**Keywords:** pigeons, homing, ECoG functional network, electrocorticogram, network topological properties

## Abstract

Pigeon homing provides an important model for studying navigation after displacement, yet how large-scale brain activity is organized across different phases of homing flight remains unclear. In this study, we recorded 16-channel epidural electrocorticographic signals from freely flying pigeons during outdoor homing flights initiated approximately 6 km from the home loft. GPS trajectories were used to divide the homing process into waiting, hovering, fuzzy positioning, precise positioning, and home stages. Correlation-based inter-channel functional networks were constructed across alpha, beta, gamma, and high-gamma frequency bands, and their topological properties were quantified using the clustering coefficient and global efficiency. The results showed that ECoG functional network topology varied across homing stages. Network measures were generally higher during the precise positioning stage near the home loft and lower during the fuzzy positioning stage. Gamma-band networks showed a relatively stronger tendency toward the precise positioning stage, whereas alpha-band networks showed a relatively stronger tendency toward the hovering stage. Together, these findings suggest that natural pigeon homing flight is accompanied by stage-associated reorganization of epidural ECoG functional networks.

## Simple Summary

Homing pigeons can return to their home loft after being released from distant locations, making them an important animal model for studying natural navigation. However, it remains difficult to record brain activity while pigeons are flying freely outdoors. In this study, we recorded epidural electrocorticographic signals from pigeons during outdoor homing flight and analyzed how the relationships among recording channels changed across different stages of the journey. The homing process was divided into waiting, hovering, fuzzy positioning, precise positioning, and home stages using position-tracking trajectory information. We found that the functional network organization of the recorded brain signals changed across these stages. In particular, network measures were generally higher during the precise positioning stage near the home loft and lower during the fuzzy positioning stage. These findings suggest that pigeon homing flight is accompanied by dynamic reorganization of large-scale brain activity, especially during the final localization phase before returning home.

## 1. Introduction

Homing is a natural navigation behavior in which an animal returns to a familiar home site after displacement. Homing pigeons, a domesticated form of Columba livia selectively bred and trained for strong homing ability, have long served as a classical model for studying orientation, spatial memory, cue integration, and route learning during free movement. Although some avian species perform seasonal migration, pigeon homing is more appropriately considered a model of navigation after displacement rather than migration. Successful homing depends on multiple environmental and internal cues, including solar information, geomagnetic cues, olfactory signals, visual landmarks, and previous spatial experience [1,2,3,4,5,6,7]. Rather than relying on a single cue system, pigeon homing is increasingly viewed as a dynamic and multimodal process in which different sources of information may contribute at different phases of the journey.

Among these sensory systems, olfactory information has played a particularly important role in the history of pigeon homing research. The olfactory-map hypothesis, developed from the work of Papi and colleagues and subsequently investigated in many behavioral studies, proposes that pigeons can use environmental odor information to establish a spatial reference map for homeward orientation [3,4]. Although the relative contribution of olfactory cues may vary with release site, familiarity, training history, and environmental conditions, converging evidence indicates that olfaction is one of the major sensory components involved in pigeon homing [3]. This olfactory perspective is important for understanding pigeon navigation as a multisensory process rather than as a behavior driven only by visual or magnetic cues.

Previous studies have provided important insights into the behavioral and sensory mechanisms of pigeon homing. GPS-based trajectory analyses have revealed route development, landmark use, individual differences in path choice, and the gradual acquisition of navigational familiarity [8,9]. Recent work has further shown that passive exposure to a novel release site alone may not be sufficient to produce detectable improvement in homing performance, suggesting that navigational learning depends on active experience and the integration of multiple forms of spatial information [10]. These behavioral findings indicate that pigeon homing is not a simple directional response but a complex natural behavior involving continuous updating of spatial and sensory information.

At the neural level, the hippocampal formation has received considerable attention because of its established role in avian spatial memory and navigation. Electrophysiological studies in homing pigeons have identified several categories of spatially related neural responses, including location-related, path-related, context-dependent, and pattern-like responses, indicating that the avian hippocampal system contributes to spatial representation in ways that are partly comparable to and partly distinct from the mammalian hippocampus [11]. Recent anatomical work has further clarified the intrinsic circuitry of the homing pigeon hippocampal formation, providing a structural basis for understanding how spatial information may be processed within this system [12]. In addition to the hippocampal formation, other forebrain structures, including the visual Wulst, have also been implicated in landmark-based homing and visually guided navigation [13]. Together, these studies suggest that pigeon homing is supported by coordinated processing across multiple neural systems rather than by a single brain region alone.

Recent outdoor electrophysiological studies have begun to move neural recordings closer to natural homing conditions. For example, hippocampal local field potential recordings during outdoor pigeon homing flight showed that neural responses differed between initial decision-making and en route navigation stages [14]. In addition, outdoor free-flight recordings from pigeons have linked gamma-band local field potential activity and local functional network dynamics to flight acceleration, suggesting that avian neural activity can vary with natural flight state [15]. These findings provide important evidence that neural activity during actual flight and homing is behaviorally and stage dependent. However, most available electrophysiological evidence remains focused on anatomically targeted recordings from specific structures or local neural populations. Less is known about how broader epidural ECoG activity is organized at the inter-channel network level during different behavioral stages of natural homing flight.

Despite this progress, directly examining large-scale neural activity during outdoor homing flight remains technically challenging. Pigeons must fly freely while carrying lightweight recording equipment, and neural signals must be preserved despite motion, wingbeat-related artifacts, environmental interference, device load, and variability in individual flight trajectories. In the present study, the release distance was approximately 6 km. This distance should not be regarded as representative of the full long-range homing capacity of highly trained racing pigeons, which can return from much greater distances under appropriate conditions. Instead, it provided a kilometer-scale outdoor homing task that allowed complete neural and trajectory recordings while maintaining electrode stability, device recovery, and comparability across trials. Such recordings provide a valuable opportunity to study neural activity under ecologically relevant but experimentally manageable free-flight conditions.

Epidural electrocorticographic recording provides a feasible approach for monitoring population-level electrophysiological activity in freely flying birds. In this approach, skull-fixed epidural electrodes record electrical signals from the dorsal cranial surface. These signals reflect the summed activity of neural populations near the recording sites and provide a mesoscopic view of electrophysiological activity during behavior. Although epidural ECoG does not provide the spatial specificity of anatomically targeted deep-brain recordings, multichannel signals can capture distributed inter-channel activity patterns during natural behavior. In this context, functional network analysis offers a useful framework for quantifying how relationships among recording channels change across behavioral stages. By constructing inter-channel correlation networks from ECoG signals and analyzing graph-theoretical properties, it is possible to characterize stage-associated changes in local clustering and global integration of large-scale electrophysiological activity [16,17,18].

In the present study, we investigated whether epidural ECoG functional networks are reorganized across different stages of natural pigeon homing flight. We recorded 16-channel epidural ECoG signals from freely flying pigeons during outdoor homing flights from a release site approximately 6 km from the home loft. GPS trajectories were used to define coarse behavioral stages of the homing process, including waiting, hovering, fuzzy positioning, precise positioning, and home. For each stage, correlation-based inter-channel functional networks were constructed across multiple frequency bands, and the clustering coefficient and global efficiency were used to quantify local and global network organization. We hypothesized that pigeon homing flight would be accompanied by stage-associated reorganization of epidural ECoG functional networks, reflecting dynamic coordination of large-scale electrophysiological activity during natural navigation.

## 2. Materials and Methods

### 2.1. Animals and Ethical Approval

Three adult domestic homing pigeons (Columba livia domestica; locally maintained for homing training), aged 2–3 years and weighing 450–550 g, were used in this study. Sex was not determined. The pigeons were numbered 027, 077, and 095 for identification. The animals were maintained in the home loft under routine husbandry conditions and were allowed to adapt to the loft environment before the experiments. Food and water were available ad libitum, and the pigeons were kept under a natural light/dark cycle. Before ECoG recording experiments, all pigeons underwent repeated homing training until they showed stable homing performance. Only pigeons that could reliably return to the home loft were selected for electrode implantation and subsequent signal acquisition. After surgery, each pigeon was temporarily housed individually for postoperative observation and was allowed to resume homing training only after full recovery.

All experimental procedures were approved by the Life Science Ethical Review Committee of Huanghuai University (approval no. 20231103003). Animal housing, surgical procedures, postoperative care, and behavioral experiments were performed in accordance with the approved institutional guidelines for animal research. No adverse events were observed during postoperative recovery or recording experiments.

### 2.2. Experimental Design and Homing Task

**Figure 1:**
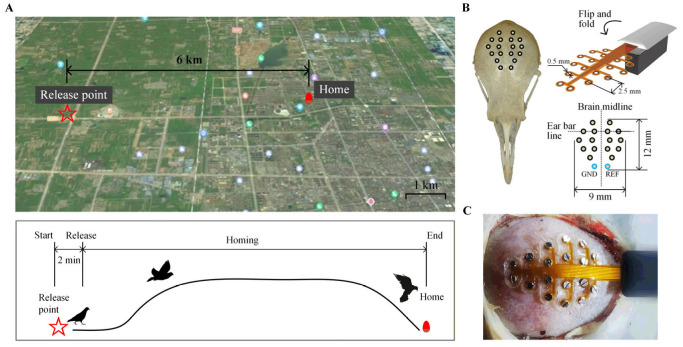
Experimental paradigm and electrode implantation. (**A**) Diagram illustrating the experimental procedure. (**B**) Electrodes and their implantation sites. (**C**) Photograph depicting electrode implantation.

The homing experiments were conducted outdoors using a release site located approximately 6 km west of the home loft. This distance was selected to provide a repeatable kilometer-scale outdoor homing task while maintaining stable ECoG recording, reliable GPS tracking, and a high probability of recovering both the animal and the recording device. Pigeons were transported to the release site and then allowed to return freely to the loft (Figure 1A). All homing tasks were conducted on sunny days after 4:00 p.m. to reduce variability associated with weather and extreme environmental conditions.

ECoG and GPS recordings were performed only after the pigeons had reached stable homing performance, defined as a homing success rate of 100% and a homing duration of less than 1 h during training. During each recording trial, ECoG signals and GPS trajectory data were acquired simultaneously. Recording began approximately 2 min before release and ended after the pigeon had entered the home loft. Only trials with complete homing behavior, recoverable ECoG signals, and usable GPS trajectory data were included in the analysis. Trials with homing durations shorter than 45 min were retained for network analysis to reduce variability associated with unusually long or interrupted flights and to improve comparability across trials. In total, six effective trials were typically obtained for each pigeon.

The outdoor free-flight setting imposed several technical constraints. Successful data acquisition required stable chronic electrode fixation, lightweight wireless recording, successful autonomous homing, and recoverable signals despite body movement, wing flapping, environmental interference, and variable flight trajectories. Therefore, each valid trial represented a complete free-flight neural recording under naturalistic conditions.

### 2.3. Surgical Implantation of Epidural ECoG Electrodes

All surgical procedures were performed under general anesthesia induced by 3% sodium pentobarbital at a dose of 0.12 mL per 100 g body weight. In addition, 2% lidocaine was administered subcutaneously as local auxiliary anesthesia. After the pain reflex had disappeared, the pigeon’s head was fixed in a custom-made stereotaxic device. The scalp was incised, and the connective tissue on the skull surface was carefully removed. Hydrogen peroxide was used to clean the skull surface, and an electrode guide film was fixed onto the skull with adhesive.

A 16-channel ECoG electrode array (Kedou Brain-Computer Technology, Suzhou, China) was fixed to the dorsal cranial surface using micro-screws with a diameter of 0.5 mm. The screws were inserted through the preformed positioning holes of the guide film according to the implantation layout shown in Figure 1B. By adjusting the implantation depth, the screw tips were brought into contact with the intracranial dura mater, enabling epidural ECoG recording from the brain surface. A representative photograph of the implanted electrode is shown in Figure 1C.

The electrode array was designed to obtain distributed surface recordings rather than to target a single deep brain structure. Therefore, the network nodes in the present study correspond to ECoG recording channels. The resulting functional networks should be interpreted as inter-channel epidural ECoG association networks, rather than as anatomically resolved connectivity between specific brain regions.

After surgery, pigeons were allowed to recover for 5–7 days before signal recording. During recovery, the general health status, feeding behavior, wound healing, and ability to fly were monitored. Recording experiments were performed only after the pigeons had recovered and resumed stable homing behavior.

### 2.4. ECoG and GPS Data Acquisition

**Figure 2:**
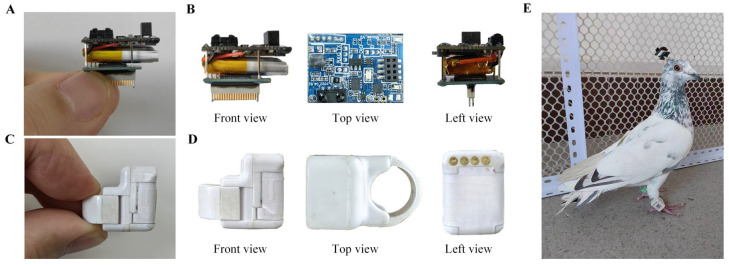
Signal acquisition equipment. (**A**) Photograph of the ECoG signal collector. (**B**) Various angles of the ECoG signal collector. (**C**) Photograph of the pigeon trajectory tracker. (**D**) Various angles of the pigeon trajectory tracker. (**E**) Pigeon equipped with signal acquisition equipment.

ECoG signals were recorded using the Hermes wireless neural signal recording system (Bio-Signal Technologies LLC, Nanjing, China). The appearance and different views of the ECoG recording device are shown in Figure 2A,B. The signals were amplified 4000 times, filtered in the range of 0–250 Hz, continuously sampled at 2 kHz, and stored on a 128 GB memory card carried by the pigeon. The recording device was fixed to the pigeon in a manner that minimized interference with natural flight.

Trajectory data were recorded using a Feiyu intelligent locator (Feiyu Technology Co., Ltd., Shenzhen, China) attached to the pigeon’s leg (Figure 2C–E). The GPS system provided position information with an accuracy of approximately 5 m and a sampling rate of 1 point per minute. This temporal resolution was sufficient to describe the overall homing trajectory and to support coarse behavioral staging, but it was not sufficient for millisecond-level alignment between neural signals and behavioral events or for precise determination of abrupt transition time points.

### 2.5. Behavioral Stage Definition

For stage-wise comparison of ECoG network organization, the homing process was divided into five coarse behavioral stages: waiting, hovering, fuzzy positioning, precise positioning, and home. These stages were operationally defined for the present study rather than adopted from a standardized ethogram. Stage definition was based on release status, GPS trajectory morphology, and the spatial relationship between the pigeon and the home loft.

The waiting stage referred to the period before release from the transport cage. The hovering stage referred to the initial post-release period, during which the pigeon typically showed local circling or clustered movement near the release site. The fuzzy positioning stage referred to the directed flight period between the release area and the home area, during which the bird generally progressed toward the loft but had not yet entered the final localization phase. The precise positioning stage referred to the final approach near the home loft, during which the trajectory became more locally adjusted and spatially concentrated around the loft area. The home stage referred to the period after the pigeon had entered the loft.

Because GPS positions were sampled once per minute, these stages were used as coarse behavioral categories rather than sharply defined behavioral states. Stage boundaries were determined by combining trajectory morphology, spatial location relative to the release site and home loft, and visual inspection of the flight path. Segments near ambiguous transitions were excluded from stage-based network analysis whenever necessary to reduce contamination between adjacent stages.

### 2.6. ECoG Preprocessing and Frequency-Band Extraction

ECoG data were analyzed using MATLAB (R2016a, MathWorks, Natick, MA, USA). The recorded signals were first inspected to identify valid recording periods corresponding to the homing process. Signal segments with severe data loss, recording interruption, or obvious nonphysiological artifacts were excluded.

For network analysis, ECoG signals were divided into four frequency bands: alpha, 8–13 Hz; beta, 13–30 Hz; gamma, 30–80 Hz; and high-gamma, 80–150 Hz. Signals in specific frequency bands were extracted using the eegfilt function in the EEGLAB toolbox (EEGLAB 2021.1). Signals below 8 Hz were not included in the functional network analysis because this low-frequency range was strongly affected by wingbeat-related electromyographic and motion interference during free flight. Signals above 150 Hz were not analyzed because they contained relatively few effective components under the present recording conditions.

Time-frequency representations were generated using a complex Morlet wavelet transform for visualization of representative ECoG activity. These time-frequency plots were used to illustrate spectral changes during homing but were not used as independent statistical samples in the functional network analysis.

### 2.7. Functional Network Construction

Functional networks were constructed separately for each pigeon, trial, homing stage, and frequency band. For each stage and frequency band, 10 s ECoG segments were selected from the corresponding stage-specific signal interval. Each segment contained M simultaneously recorded ECoG channels. In the present study, functional connectivity refers to the correlation structure among ECoG channels within a given time segment, rather than to a direct measure of causal interaction or anatomically resolved connectivity between brain regions.

For each 10 s segment, pairwise Pearson correlation coefficients were calculated between all channel pairs. For two channels *i* and *j*, the Pearson correlation coefficient was calculated according to Equation (1):

(1) $$r_{ij}=\frac{\sum_{t=1}^{T}\left[x_{i}\left(t\right)-{\bar{x}}_{i}\right]\left[x_{j}\left(t\right)-{\bar{x}}_{j}\right]}{\sqrt{\sum_{t=1}^{T}{\left[x_{i}\left(t\right)-{\bar{x}}_{i}\right]}^{2}\sum_{t=1}^{T}{\left[x_{j}\left(t\right)-{\bar{x}}_{j}\right]}^{2}}}$$

where $x_{i}\left(t\right)$ and $x_{j}\left(t\right)$ denote the filtered ECoG signals from channels *i* and *j* at time point *t*, ${\bar{x}}_{i}$ and ${\bar{x}}_{j}$ denote their mean values within the 10 s segment, and *T* denotes the number of samples in the segment.

The pairwise correlation coefficients across all channel pairs were then assembled into an M×M functional connectivity matrix according to Equation (2):

(2) $$R=\left[\begin{array}{cccc} r_{1,1} & r_{1,2} & \ldots & r_{1,M}\\ r_{2,1} & r_{2,2} & \ldots & r_{2,M}\\ \vdots & \vdots & \ddots & \vdots \\ r_{M,1} & r_{M,2} & \ldots & r_{M,M} \end{array}\right]$$

where ***R*** denotes the functional connectivity matrix, *M* denotes the number of ECoG channels, and $r_{ij}$ denotes the Pearson correlation coefficient between channels *i* and *j*. The diagonal elements $r_{ij}$ were equal to 1 in the correlation matrix.

To facilitate comparison of network topology across homing stages and frequency bands, the correlation matrix was converted into a binary adjacency matrix using a fixed threshold. Correlation coefficients greater than 0.95 were assigned a value of 1, whereas correlation coefficients less than or equal to 0.95 were assigned a value of 0. The diagonal elements were set to 0 to remove self-connections. The binary adjacency matrix was defined according to Equation (3):

(3) $$a_{ij}=\{\begin{array}{cc} 1, & r_{ij}>0.95,\;i\neq j,\\ 0, & r_{ij}\leq 0.95,\;i\neq j,\\ 0, & i=j. \end{array}$$

where $a_{ij}$ denotes the connection state between channels *i* and *j* in the binary network. When $a_{ij}=1$, a suprathreshold positive correlation was considered to exist between the two channels; when $a_{ij}=0$, no suprathreshold connection was assigned.

The same threshold was applied across all frequency bands and homing stages. A stringent threshold of 0.95 was used to emphasize the strongest inter-channel associations and to reduce the influence of weak or unstable correlations during outdoor free-flight recording. The resulting binary network therefore represents the high-correlation inter-channel structure of ECoG activity within each stage and frequency band. Because graph-theoretical measures can depend on threshold selection, the network results should be interpreted as threshold-dependent properties of inter-channel ECoG association patterns rather than as absolute measures of anatomical or causal connectivity.

### 2.8. Graph-Theoretical Network Measures

To characterize stage-associated changes in epidural ECoG functional network topology, two graph-theoretical measures were calculated from the thresholded binary networks: clustering coefficient and global efficiency. These two measures were selected because they describe complementary aspects of network organization. The clustering coefficient was used to quantify local inter-channel clustering, whereas global efficiency was used to quantify global topological integration of the network.

For each binary network, the clustering coefficient was first calculated at the node level. For node (i), the clustering coefficient was defined as:

(4) $$C_{i}=\frac{2e_{i}}{k_{i}\left(k_{i}-1\right)}$$

where $k_{i}$ denotes the degree of node *i*, and $e_{i}$ denotes the number of existing edges among the neighbors of node *i*. If $k_{i}<2$, $C_{i}$ was defined as 0. A higher $C_{i}$ indicates that the neighboring nodes of node *i* are more strongly interconnected.

The average clustering coefficient of the whole network was then calculated as:

(5) $$C=\frac{1}{N}\sum_{i=1}^{N}C_{i}$$

where *N* denotes the number of nodes in the network. In the present study, each node corresponded to one epidural ECoG recording channel. Therefore, the average clustering coefficient was interpreted as a channel-level index of local inter-channel organization in the epidural ECoG functional network.

Global efficiency was used to describe the global topological integration of the binary network. It was calculated as:

(6) $$E_{glob}=\frac{1}{N\left(N-1\right)}\sum_{i\neq j}\frac{1}{d_{ij}}$$

where $d_{ij}$ denotes the shortest path length between nodes *i* and *j*. If no path existed between two nodes, $1/d_{ij}$ was defined as 0. A higher global efficiency indicates shorter graph-theoretical distances among nodes and stronger global integration of the binary inter-channel network.

Both clustering coefficient and global efficiency were calculated for each sampled 10 s segment and then averaged within each trial, homing stage, and frequency band. Because the network nodes represented epidural ECoG recording channels rather than anatomically defined brain regions, these graph-theoretical measures were interpreted as channel-level network properties. They should not be interpreted as direct measures of anatomical connectivity or causal information transfer between specific brain structures.

### 2.9. Resampling Procedure and Statistical Analysis

Because the durations of the behavioral stages were not identical across trials, a resampling procedure was used to obtain comparable network estimates. For each trial, homing stage, and frequency band, 10 s ECoG segments were randomly sampled 50 times from the corresponding stage interval. Network construction and graph measure calculation were performed for each sampled segment. The resulting 50 values were then averaged to obtain a single trial-level estimate for that stage and frequency band.

The 50 sampled windows were used as within-trial resampling estimates and were not treated as independent biological observations in the final stage-wise comparisons. The trial-level estimates were used for statistical analysis and group-level visualization. Unless otherwise stated, data are presented as mean ± standard deviation.

Given the limited sample size and the absence of assumptions regarding normality, stage-wise differences in network measures were evaluated using the nonparametric Wilcoxon rank-sum test. The significance level was set at 0.05. Statistical analyses were performed using the MATLAB Statistics and Machine Learning Toolbox. The statistical results were used to evaluate stage-associated differences in ECoG network topology and should be interpreted in the context of the technically challenging outdoor free-flight recording conditions.

## 3. Results

### 3.1. Homing Trajectories and Behavioral Stage Definition During Free Flight

To investigate stage-associated reorganization of epidural ECoG functional networks during natural homing flight, ECoG signals and GPS trajectories were recorded simultaneously from freely flying pigeons during outdoor homing trials. After being transported to a release site approximately 6 km from the home loft, the pigeons returned autonomously to the loft. During successful trials, the animals typically showed a characteristic sequence of behavioral phases, including a pre-release waiting period, an initial post-release flight period near the release site, a directed flight segment toward the home area, a final approach near the loft, and the post-arrival home period.

**Figure 3:**
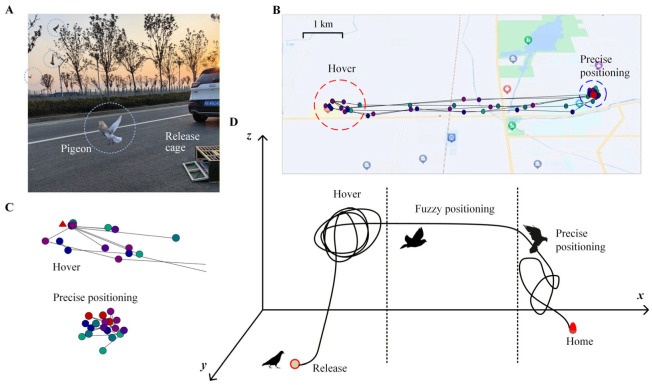
Pigeon trajectories during homing. (**A**) Photograph showing pigeon release. (**B**) Trajectories of pigeons during homing in different trials (n = 6). Different colors indicate the six individual homing trials, and dots represent GPS-recorded positions along the trajectories, with a time interval of 1 min between points. (**C**) Enlarged segments of pigeon trajectories during hovering and precise positioning (n = 6), corresponding to positions marked by the red and blue dashed lines in (**B**), respectively. (**D**) Schematic diagram illustrating the division of different stages during homing.

Representative homing trajectories are shown in Figure 3. The pigeons did not return along a completely uniform path. Instead, the trajectories typically included local circling or clustered movement near the release site, followed by a more directed flight segment, and finally local adjustments near the home loft. Based on release status, trajectory morphology, and the spatial relationship between the pigeon and the home loft, the homing process was operationally divided into five coarse behavioral stages: waiting, hovering, fuzzy positioning, precise positioning, and home.

The waiting stage referred to the period before release from the transport cage. The hovering stage referred to the initial post-release period, during which the pigeon showed local movement near the release site. The fuzzy positioning stage referred to the main directed flight segment between the release area and the home area. The precise positioning stage referred to the final approach near the loft, during which the trajectory became more locally adjusted around the home area. The home stage referred to the period after the pigeon had entered the loft.

Because the GPS sampling rate was 1 point per minute, these stages were used as coarse behavioral categories rather than sharply defined behavioral states. This stage definition provided a behaviorally interpretable framework for comparing epidural ECoG functional network organization across different phases of the homing process.

### 3.2. Epidural ECoG Signals Recorded During Pigeon Homing Flight

**Figure 4:**
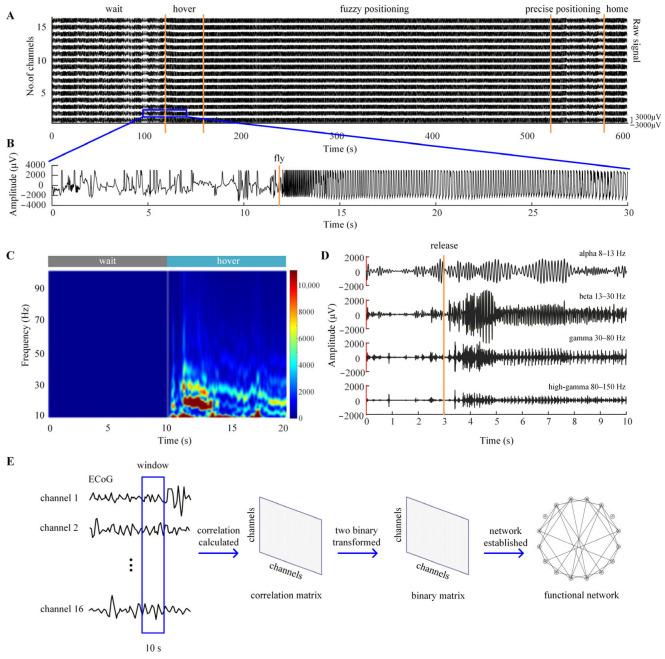
ECoG signals recorded during pigeon homing. (**A**) Example of ECoG signals during a representative trial. The orange line, together with the trajectories, indicates the positions of different stages. (**B**) Enlarged view of the ECoG signal highlighted by the blue box in (**A**). (**C**) Time-frequency spectrum of the ECoG signal highlighted by the blue box in (**A**). (**D**) Examples of rhythmic ECoG signals in different frequency bands: alpha (8–13 Hz), beta (13–30 Hz), gamma (30–80 Hz), and high-gamma (80–150 Hz), from top to bottom. (**E**) Flowchart illustrating the construction of the inter-channel ECoG functional network using ECoG signals. The release time is indicated by the orange line in (**B**–**D**).

Continuous multichannel epidural ECoG signals were recorded during outdoor homing flight. Representative ECoG signals from a successful homing trial are shown in Figure 4A. The recordings covered the waiting period, release, in-flight homing stages, and the home stage after arrival at the loft. These recordings confirmed that epidural ECoG signals could be obtained throughout the natural homing process under outdoor free-flight conditions.

An enlarged segment of the ECoG signal showed that the low-frequency range was strongly affected by wingbeat-related electromyographic and motion interference during active flight (Figure 4B). Therefore, subsequent functional network analysis focused on frequency components above 8 Hz. Time-frequency visualization further showed that ECoG spectral activity changed around and after release (Figure 4C).

To examine functional network organization across different oscillatory ranges, ECoG signals were divided into four frequency bands: alpha, beta, gamma, and high-gamma. Representative filtered traces from these frequency bands are shown in Figure 4D. These signals showed frequency-band-specific rhythmic activity patterns during homing, providing the basis for subsequent stage-specific network construction.

### 3.3. Construction of Stage-Specific Epidural ECoG Functional Networks

To characterize inter-channel network organization during homing, correlation-based functional networks were constructed separately for each homing stage and frequency band. The network construction procedure is summarized in Figure 4E. For each selected 10 s ECoG segment, pairwise Pearson correlation coefficients were calculated between all ECoG channels to obtain a functional connectivity matrix. The correlation matrix was then converted into a binary adjacency matrix using a fixed threshold, and the resulting network was used for graph-theoretical analysis.

**Figure 5:**
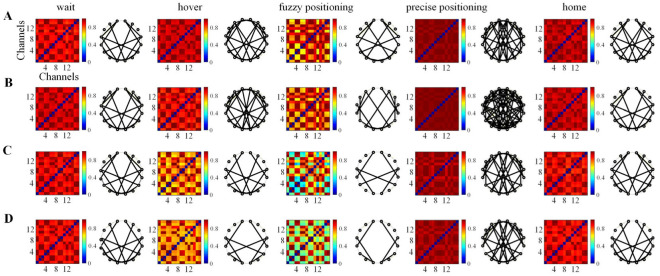
Functional networks of different homing stages constructed from different ECoG rhythm signals (pigeon 027 shown as an example). (**A**) Functional network constructed from the alpha band. (**B**) Functional network constructed from the beta band. (**C**) Functional network constructed from the gamma band. (**D**) Functional network constructed from the high-gamma band. From left to right: waiting, hovering, fuzzy positioning, precise positioning, and home. For each stage, the left panel shows the correlation coefficient matrix, and the right panel shows the binary functional network.

Representative stage-specific functional networks are shown in Figure 5. Across the alpha, beta, gamma, and high-gamma bands, the structure of the correlation matrices and binary networks varied across homing stages. In general, the fuzzy positioning stage showed relatively sparse high-correlation inter-channel network patterns, whereas the precise positioning stage showed denser network patterns. These visual differences indicated that the organization of inter-channel epidural ECoG associations was not constant throughout homing flight.

Because the electrode array was placed on the dorsal cranial surface and recorded epidural signals, the networks shown in Figure 5 should be interpreted as inter-channel ECoG functional networks rather than anatomically resolved connectivity maps between specific brain regions. The visual stage-related differences in Figure 5 motivated further quantitative comparison of network topology across homing stages.

### 3.4. Stage-Associated Changes in Local Network Clustering

To quantify local network organization, the clustering coefficient was calculated from the thresholded binary networks. The clustering coefficient reflects the extent to which neighboring nodes form locally interconnected structures and was used here as an index of local inter-channel organization in the epidural ECoG functional network.

**Figure 6:**
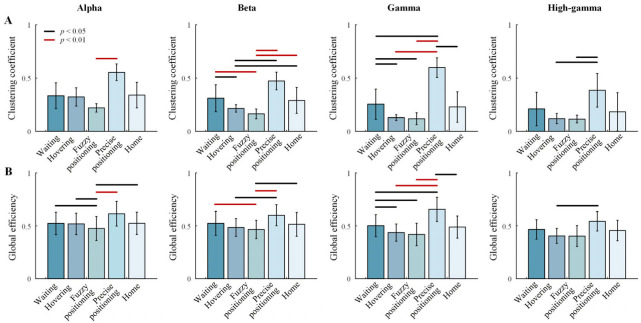
Stage-associated changes in ECoG functional network topology across frequency bands. (**A**) Clustering coefficient. (**B**) Global efficiency. From left to right, the frequency bands are alpha, beta, gamma, and high-gamma. Bars represent mean ± standard deviation. Horizontal lines indicate statistically significant pairwise differences between the connected homing stages. Black lines indicate (*p* < 0.05), and red lines indicate (*p* < 0.01). Pairs without horizontal lines showed no significant difference.

Across the four frequency bands, the clustering coefficient showed stage-associated variation (Figure 6A). In general, the precise positioning stage showed higher clustering coefficient values than the fuzzy positioning stage. This pattern indicates that local clustering of inter-channel ECoG functional networks was stronger during the final localization phase near the home loft. Significant pairwise differences were observed in multiple frequency bands, as indicated by the horizontal lines in Figure 6A.

The stage-related pattern was most prominent in comparisons involving the fuzzy positioning and precise positioning stages. During fuzzy positioning, clustering coefficient values were relatively low, suggesting weaker local inter-channel clustering during the main directed flight segment. During precise positioning, clustering coefficient values increased, suggesting enhanced local coordination of inter-channel epidural ECoG activity when pigeons approached the home loft and performed final localization. These results indicate that local properties of ECoG functional networks are reorganized across homing stages.

### 3.5. Stage-Associated Changes in Global Network Integration

To evaluate global network integration, global efficiency was calculated from the same thresholded binary networks. Global efficiency reflects the inverse of shortest path length across the network and was used here as a graph-theoretical index of global inter-channel integration.

Global efficiency also showed stage-associated variation across homing phases (Figure 6B). Similar to the clustering coefficient, global efficiency tended to be lower during fuzzy positioning and higher during precise positioning. Significant pairwise differences were observed in several frequency bands, as indicated by the horizontal lines in Figure 6B.

The increase in global efficiency during precise positioning suggests that the binary inter-channel ECoG network became more globally integrated during the final approach to the home loft. In contrast, lower global efficiency during fuzzy positioning indicates a comparatively less integrated network topology during the directed flight segment. Although clustering coefficient and global efficiency quantify different aspects of network organization, both measures showed a broadly consistent stage-associated pattern. The parallel changes in these two measures support the presence of stage-dependent shifts in both local and global network topology.

### 3.6. Frequency-Band Tendencies in Functional Network Organization

**Figure 7:**
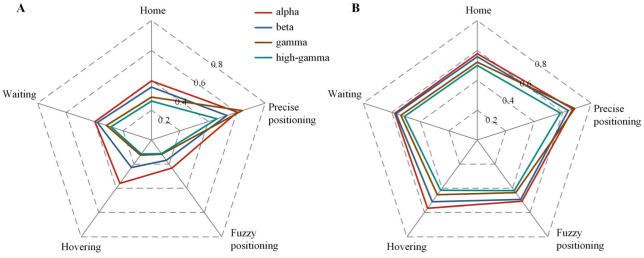
Radar map of network topological properties. (**A**) Clustering coefficient. (**B**) Global efficiency.

To further examine frequency-band-related tendencies in network organization, the clustering coefficient and global efficiency were summarized across homing stages using radar plots (Figure 7). Across alpha, beta, gamma, and high-gamma networks, both graph-theoretical measures showed stage-related variation, indicating that the reorganization of epidural ECoG functional networks was not restricted to a single narrow frequency band.

Compared with global efficiency, the clustering coefficient showed a less uniform distribution across frequency bands. Gamma-band networks appeared to show a relatively stronger preference for the precise positioning stage, whereas alpha-band networks appeared to show a relatively stronger preference for the hovering stage. These results suggest that different oscillatory ranges may be associated with different stage-related patterns of epidural ECoG network organization during homing.

These frequency-band tendencies should be interpreted as band-related network patterns rather than definitive evidence for frequency-specific neural mechanisms. Nevertheless, together with the stage-wise results in Figure 6, the radar plots further support the central observation that epidural ECoG functional networks are dynamically reorganized across different stages of pigeon homing flight.

## 4. Discussion

In this study, we investigated stage-associated reorganization of epidural ECoG functional networks during natural pigeon homing flight. By combining outdoor free-flight ECoG recordings with GPS-based behavioral staging, we constructed inter-channel functional networks across different homing stages and frequency bands. The main finding was that ECoG functional network topology varied across the homing process. In particular, both clustering coefficient and global efficiency tended to be higher during the precise positioning stage and lower during the fuzzy positioning stage. These results suggest that epidural ECoG network organization varies with behavioral phase during natural homing flight.

Pigeon homing is a complex natural behavior that requires continuous integration of multiple types of information. During the journey from a release site to the home loft, pigeons may rely on olfactory cues, geomagnetic information, solar cues, visual landmarks, and learned spatial experience [1,2,3,4,5,6,7]. The olfactory-map hypothesis further emphasizes that environmental odor information can contribute to the formation of a spatial reference map for homeward orientation [3,4]. Behavioral and GPS-based studies have shown that homing pigeons can develop familiar routes, show route fidelity, combine compass and landmark information, and adjust their trajectories according to environmental and experiential factors [8,9,10,19,20,21]. These behavioral findings imply that homing is not a simple directional response, but a dynamic navigation process involving changing behavioral demands across the journey. The present results extend this view by showing that epidural ECoG functional network organization also changes across behaviorally defined homing stages.

The relatively higher clustering coefficient observed during the precise positioning stage suggests that local inter-channel network organization was strengthened when pigeons approached the home loft. The precise positioning stage corresponded to the final approach near the loft, during which the trajectory became more locally adjusted around the home area. This behavioral stage may require stronger integration of familiar visual landmarks, spatial memory, sensorimotor feedback, and final orientation toward the loft entrance. Previous studies have emphasized the role of the avian hippocampal formation in spatial memory, large-scale spatial representation, and homing-related navigation [11,12,22,23]. In addition, the visual Wulst has been implicated in familiar landmark-based navigation, suggesting that visually guided homing may involve forebrain structures beyond the hippocampal formation [13]. From a graph-theoretical perspective, a higher clustering coefficient indicates that neighboring nodes tend to form more locally interconnected structures [16,17,24]. In the present study, this result suggests enhanced local coordination among epidural ECoG recording channels during the final localization phase of homing.

Global efficiency showed a broadly similar stage-associated pattern. Compared with the fuzzy positioning stage, the precise positioning stage generally showed higher global efficiency, indicating stronger global topological integration of the binary inter-channel ECoG network. Global efficiency reflects the inverse of shortest path length across the network and is commonly used as an index of large-scale network integration [16,17,18,25]. In the present behavioral context, the increase in global efficiency during precise positioning may reflect stronger distributed coordination of electrophysiological activity when pigeons are close to the home loft and need to complete final spatial localization. Importantly, clustering coefficient and global efficiency quantify different aspects of network topology. The convergence of these two measures strengthens the conclusion that pigeon homing is accompanied by coordinated changes in both local clustering and global integration of epidural ECoG functional networks.

In contrast, the fuzzy positioning stage showed relatively lower clustering coefficient and global efficiency. This stage corresponded to the main directed flight segment between the release area and the home area, during which pigeons generally moved toward the loft but had not yet entered the final localization phase. Compared with precise positioning, this stage may involve sustained route progression with less local adjustment around the target area. The lower network measures during fuzzy positioning therefore suggest a comparatively less clustered and less globally integrated inter-channel ECoG network structure. This does not necessarily imply reduced neural activity or lower behavioral demand. Rather, it indicates that the correlation-based network topology of epidural ECoG signals differed from that observed during final positioning. Thus, different homing stages may be associated with different modes of large-scale electrophysiological coordination.

The frequency-band analysis further suggested that stage-associated network reorganization was not restricted to a single oscillatory range. Across alpha, beta, gamma, and high-gamma bands, network measures showed stage-related variation. Gamma-band networks appeared to show a relatively stronger preference for the precise positioning stage, whereas alpha-band networks appeared to show a relatively stronger preference for the hovering stage. Recent outdoor electrophysiological recordings have shown that hippocampal LFP responses differ between initial decision-making and en route navigation stages during pigeon homing flight [14]. Another outdoor free-flight study reported that gamma-band activity and local functional network properties in the avian AId region varied with flight acceleration, suggesting that neural activity and local network organization can change with natural flight state [15]. In this context, the present frequency-band patterns are consistent with the view that different oscillatory components may participate in stage-associated network organization during homing. However, these band-related tendencies should not be interpreted as definitive evidence that specific frequency bands implement specific homing functions. They are better regarded as frequency-dependent network patterns that accompany different behavioral stages.

A key feature of the present study is that ECoG signals were recorded during outdoor free-flight homing behavior. Neural recordings under such conditions are technically demanding. Pigeons must carry a lightweight recording device, maintain stable electrode contact, complete autonomous homing behavior, and generate recoverable signals despite body movement, wingbeat-related interference, environmental variability, and device load. These constraints make stable datasets from natural homing flight difficult to obtain. Although the number of animals was limited, each valid trial represented a complete outdoor homing flight with simultaneous ECoG and GPS data. Therefore, the dataset provides electrophysiological observations from an ecologically relevant behavioral context that is difficult to reproduce under laboratory conditions. This naturalistic setting is an important complement to controlled studies of avian spatial cognition, neural representation, and navigation [11,14,15,26]. These data may also provide preliminary biological inspiration for future state-dependent or bio-inspired navigation models, although direct translation to artificial navigation systems or neural-network algorithms will require larger datasets and dedicated computational modeling.

Several methodological boundaries should be considered when interpreting the present results. First, the number of pigeons was limited because outdoor free-flight ECoG recording requires chronic electrode stability, lightweight wireless recording, reliable GPS tracking, and successful recovery of both the animal and the recording device. Therefore, the present findings should be regarded as evidence from a technically demanding exploratory dataset rather than as a complete characterization of population-level variability in homing pigeons. Second, the release distance was approximately 6 km. Although this distance was sufficient for obtaining complete outdoor ECoG and GPS recordings across distinct behavioral stages, it does not represent the full homing capacity of highly trained racing pigeons. Future studies using larger samples and longer release distances will be needed to test whether similar stage-associated ECoG network patterns can be observed under broader homing conditions.

Additional methodological considerations relate to signal interpretation and network construction. Third, the electrode array was fixed on the skull surface and recorded epidural signals over a broad dorsal cranial area. Therefore, the network nodes represented recording channels located at different epidural recording sites rather than anatomically defined brain regions. The observed networks should not be interpreted as direct connectivity between specific avian brain structures. Fourth, functional connectivity was estimated using Pearson correlation and then converted into a binary network using a fixed threshold. This approach emphasizes strong ECoG associations and allows stage-wise comparison of graph measures, but the resulting network properties are threshold-dependent [17,27,28,29,30,31]. Fifth, GPS data were sampled at 1 point per minute, which was sufficient for coarse trajectory-based staging but not for precise alignment between neural dynamics and rapid behavioral transitions. Thus, the five-stage framework should be regarded as an operational classification for this study rather than a standardized ethogram for pigeon homing. Finally, signals below 8 Hz were excluded because they were strongly affected by wingbeat-related motion and electromyographic interference during free flight. Future studies with higher-resolution behavioral tracking, improved artifact suppression, weighted-network or multi-threshold analyses, and more anatomically targeted recordings will be needed to further clarify the circuit mechanisms underlying avian homing.

Despite these limitations, the present study provides evidence that natural pigeon homing flight is accompanied by stage-associated reorganization of epidural ECoG functional networks. The precise positioning stage was characterized by relatively higher clustering coefficient and global efficiency, suggesting stronger local clustering and global integration of inter-channel electrophysiological activity during final localization near the home loft. These findings support the idea that large-scale neural activity organization changes dynamically as pigeons progress through different stages of natural navigation. By demonstrating functional network reorganization during outdoor free-flight homing, this study contributes to a broader understanding of the neural basis of natural animal navigation.

## 5. Conclusions

In this study, we recorded epidural ECoG signals from freely flying pigeons during outdoor homing flight and constructed correlation-based epidural ECoG functional networks across different homing stages and frequency bands. The results showed that ECoG functional network topology varied across the homing process, indicating that the organization of epidural electrophysiological activity was not constant during natural navigation. In particular, the precise positioning stage was characterized by relatively higher clustering coefficient and global efficiency, whereas the fuzzy positioning stage showed relatively lower network measures. These findings suggest that pigeon homing flight is accompanied by stage-associated reorganization of epidural ECoG functional networks, with stronger local clustering and global integration during the final localization phase near the home loft. Because the network nodes represented ECoG recording channels located at different epidural recording sites, the present results should be interpreted as correlation-based epidural ECoG network patterns rather than as anatomically resolved connectivity between specific brain regions. This study provides electrophysiological evidence that large-scale neural activity organization changes dynamically during natural homing behavior and offers a useful basis for future investigations combining outdoor free-flight neural recording, higher-resolution behavioral tracking, and more detailed circuit-level analyses to further clarify the neural mechanisms underlying avian navigation.

## Data Availability

The data supporting the findings of this study are available from the corresponding author upon reasonable request. The raw electrophysiological recordings and GPS trajectory data are not publicly available because they are part of ongoing analyses and require additional experimental context and preprocessing information for appropriate interpretation.
